# Investigation into potential mechanisms of metabolic syndrome by integrative analysis of metabolomics and proteomics

**DOI:** 10.1371/journal.pone.0270593

**Published:** 2022-07-05

**Authors:** Meimei Chen, Zhaoyang Yang, Huijian Gan, Yang Wang, Chandong Li, Yuxing Gao

**Affiliations:** 1 College of Traditional Chinese Medicine, Fujian University of Traditional Chinese Medicine, Fuzhou, Fujian, China; 2 Fujian Key Laboratory of TCM Health Status Identification, Fujian University of Traditional Chinese Medicine, Fuzhou, Fujian, China; 3 Engineering Research Center, Fujian University of Traditional Chinese Medicine, Fuzhou, Fujian, China; 4 College of Chemistry and Chemical Engineering, Xiamen University, Xiamen, Fujian, China; Queen’s University Belfast, UNITED KINGDOM

## Abstract

Metabolic syndrome (MetS) is a complex syndrome cluster of metabolic disorders, which greatly increases the risks of diabetic and cardiovascular diseases. Although it has become a significantly worldwide public health burden, its pathogenesis largely remains unknown. In this study, we first performed an integrated analysis of proteomic and metabonomic data of liver tissues of rats between MetS and control groups to reveal possible mechanisms of MetS. A total of 16 significantly perturbed pathways were identified, of which three pathways were shared by patients with MetS and diabetes identified by analysis of serum samples, including alanine, aspartate and glutamate metabolism, valine, leucine and isoleucine biosynthesis, and glycine, serine and threonine metabolism. Additionally, it was found that 18 differential metabolites were closely related with 36 differential proteins, which were considered as significantly discriminant metabolites and proteins between two groups and were mainly involved in metabolic processes of gamma-aminobutyric acid and acetyl-CoA, biosynthetic processes of cholesterol and amino acids. The results of PPI network analysis and topological parameter calculation of four methods revealed that 16 proteins can serve as hub proteins of MetS. Followed by searching the PubMed database and molecular docking of Cyp7a1 and Got1, we concluded that atorvastatin and resveratrol may be potential drugs for MetS.

## Introduction

Metabolic syndrome (MetS) describes a cluster of disorders that include central obesity, hypertension, dyslipidemia and impaired glucose tolerance, which lead to the development of type 2 diabetes mellitus (T2DM) and cardiovascular diseases. Nowadays, it is estimated that 25% of adults worldwide suffer from MetS and is recognized as a global public health concern [1]. Insulin resistance and energy imbalance are widely regarded to be the primary causes of MetS [2]. Although the roles of the two main causes in MetS are plausible, its specific pathogenesis still remains unknown and it also lacks screening and optimal treatment for this disease. Currently, proteomics and metabonomics have been promising technologies for elucidating the mechanisms of diseases, which detect and identify various molecules at the levels of proteins and metabolites, and study their functions and interrelationships among various molecules [3, 4]. Yu Yuan et al. combined the two techniques to explore the mechanism of doxorubicin-induced heart failure in rats and found that PTP1B can be used as a potential target for the treatment of heart failure [3]. In recent years, some metabonomics studies have been used to detect changing levels of serum metabolites of MetS. Because MetS is a complex disease related to gene factors and external environment (diet, lifestyle), and lacks for unique diagnostic criteria, there is still no uniform statement on the biomarkers of MetS, which mostly have involved the abnormal metabolism of amino acids so far [5]. Alanine has been reported to be related to several characteristics associated with MetS, including body mass index (BMI), waist circumference (WC), triglycerides, hypertension, impaired glucose tolerance, and insulin resistance [6]. The branch-chain amino acids including isoleucine, leucine, and valine are closely related to metabolic diseases. A 2018 meta-analysis of four groups of T2DM patients showed that these three branched chain amino acids increased by approximately 40% in the case of poor blood glycemic control [7]. These studies have some implications for MetS, but they are stayed at the metabolite level. Given that proteins are the main performers of life activities and that alterations in protein expression levels are directly related to disease, drug action or toxin action, it is clear that if metabolomics and proteomics are integrated, it will provide more possibilities to understand the underlying molecular mechanisms of MetS.

Considering that the liver is the main organ of metabolism in the body and the metabolism center of various substances, it plays an important role in the metabolism of the three major nutrients of carbohydrates, lipids and proteins. It involves many functions such as synthesis, storage, decomposition, excretion, detoxification and secretion. Thus, liver metabolism likely plays a significant role in metabolic diseases. An analysis of the physical examination data of 21928 Chinese elderly people over 65 years found that the risk of metabolic syndrome in the abnormal liver function group is 1.948 times that of the normal liver function group, and all the main components of metabolic syndrome such as high blood glucose, high triglycerides, overweight or obesity were closely related to abnormal liver function [8]. Additionally, Amedeo Lonardo et al., concluded that nonalcoholic fatty liver disease preceded and was a risk factor for the future development of the metabolic syndrome based on 19 longitudinal studies [9].

Thus, it can be concluded that changes in levels of metabolites and proteins of liver tissues can be used to reveal possible mechanisms of MetS, which can reflect molecular processes closer to the disease state than serum samples. Since MetS is caused by multi-factors including genetic and environmental factors, animal modeling can minimize the differences between these two main factors, and is more suitable for preliminary studies of common mechanisms of MetS in human. At present, MetS modeling methods are mostly induced by diet such as high-sugar, high-fat diet or high-sugar/high-fat diet. The advantage of the diet-induced model is that it can simulate environmental factors such as restricting its activities, the modelling rate is high, and the model is close to the clinic [10]. In this study, high-throughput analysis technologies including gas chromatography/time-of-flight mass spectrometry (GC-TOF/MS) and high performance liquid chromatography-tandem mass spectrometry (HPLC-MS/MS) combined with KEGG (Kyoto Encyclopedia of Genes and Genomes) pathway enrichment analysis were performed to detect the differential metabolites and proteins of liver tissues of MetS model rats to explore potential proteins, pathways and drugs for the prevention and treatment of MetS.

## Materials and methods

### Chemicals

Main chemicals including L-2-chlorophenylalanine (Shanghai Hengbai Biotechnology Inc. China), saturated fatty acid methyl esters (FAMEs: C8, C9, C10, C12, C14, C16, C18, C20, C22, C24) (Dr. Ehrenstorfer GmbH, Germany), formic acid (Fisher Scientific, USA), acetonitrile (75–05-8, Fisher Scientific, USA), BCA Kit (Fisher Scientific, USA), trifluoroacetic acid (Fisher Scientific, USA), protease inhibitor cocktail (Roche, Switzerland), Na-upgrader peptide column (Thermo, USA), trypsin (Promega, Madison, WI), were used.

### Animals

This study was approved by the Experimental Animal Ethical Committee of Fujian University of Traditional Chinese Medicine, Fuzhou, P. R. China (FUTCMME No. 020/2016), and all experiments were performed strictly according to the Guide for the Care and Use of Laboratory Animals (US National Research Council 1996). Liver tissues of Wistar rats in the normal control and MetS model groups (seven rats in each) were taken from our lab at random [11]. Here, the adult male Wistar rats (2 months) weighing 200±20 g were used in the MetS modeling experiment, and the specific information of housing conditions can be seen in the supplemental information. Rats in the normal control group were fed with normal diet for 17 weeks, and rats in the MetS model group were fed with fifteen-week’s high-sugar-fat-diet and two-week’s high-fat emulsion [S1 File]. The metabolic disorder of the animals was assessed by measuring abdominal perimeters, serum levels of HDL-C and insulin, and insulin-resistances (HOMA-IR, estimated using the homeostasis model assessment) according to the WHO definition [12]. After 17 weeks, all these parameters of MetS group were significantly different from that of normal control group, the specific result can be seen in S1 Fig in S1 File. These rats were anesthetized with an intraperitoneal injection of 10% chloral hydrate (0.3 mL/100g body weight), the livers were quickly removed and stored in -80°C. Here, metabonomics and proteomics technologies were integrated to analyze differential metabolites and proteins of the liver tissues of normal control and MetS groups.

### Metabonomics analysis

The liver tissue sample of 50±1 mg from each rat was transferred into a 2 mL tube, and 450 μL pre-cold extraction mixture (methanol/chloroform (v:v) = 3:1) with 10 μL internal standard (L-2-Chlorophenylalanine, 1 mg/mL stock) were added and centrifuged at 12 000 rpm for 15 min at 4°C. Then, 200 μL of the supernatant was transferred into a new tube, and simultaneously 60 μL of each supernatant was taken out and pooled as quality control samples (QC samples), respectively. After evaporation in a vacuum concentrator, 30 μL of methoxyamination hydrochloride (20 mg/mL in pyridine) were put into the extracts, followed by the incubation at 80°C for 30 min. Afterwards, 40 μL of the BSTFA regent (1% TMCS, v/v) was added to samples and then incubated at 70°C for 1.5 h. Finally, 5 μL of FAMEs (in chloroform) was added to each QC sample.

Then, an Agilent 7890 gas chromatograph system combined with a time-of-flight mass spectrometer was carried out to perform the GC-TOF/MS analysis for liver samples [13]. A DB-5MS capillary column (30 m×250 μm i.d., 0.25 μm film thickness; J&W Scientific, Folsom, CA, USA) was applied for all analytes. The initial temperature of column was maintained at 50°C for 1 min, and then gradually increased to 310°C at a rate of 10°C/min, and stayed at 310°C for 8 min. Subsequently, we controlled the temperatures of injection, transfer line and ion source at 280°C, 280°C and 250°C, respectively. The electron energy was -70 eV. Finally, the mass spectrometry data were gained at a rate of 12.5 spectra/second with a solvent delay of 6.25 min from the m/z range of 50 to 500 in a full-scan mode.

### Quantitative proteomics analysis

#### Protein extraction

Rat liver tissue samples were frozen with liquid nitrogen, ground, and crushed with 1.0 milliliter of lysis buffer (7M urea, 4% SDS, 1x Protease Inhibitor Cocktail), followed by sonication on ice and centrifugation at 13000 rpm for 10min at 4°C. Protein quantification was performed by bicinchoninic acid (BCA) method. 100 μg protein per condition was transfered, alkylated and digested in the centrifugal unit. After digested with sequence-grade modified trypsin (Promega, Madison, WI) at 37°C for 12 hours, the resultant peptide mixture was labeled using chemicals from the iTRAQ8Plex reagent kit (AB Sciex, CA, USA) according to the manufacturer’s protocol (S1 File). The labeled samples were combined, desalted using C18 SPE column (Sep-Pak C18, Waters, Milford, MA) and dried in vacuo.

#### High pH reverse phase separation

The peptide mixture was re-dissolved in buffer A (buffer A: 10 mM aqueous ammonium formate, pH 10.0, adjusted with ammonium hydroxide), and then separated by high pH using the Aquity UPLC system (Waters Corporation, Milford, MA) connected to a reverse phase column (BEH C18 column, 2.1 mm x 150 mm, 1.7 μm, 300 Å, Waters Corporation, Milford, MA). The linear gradients were used for high pH separations from 0% B to 45% B in 35 minutes (B: 10 mM ammonium formate in 90% acetonitrile, pH 10.0, adjusted with ammonium hydroxide). The column flow rate was set at 250 μL/min, and the column temperature was fixed at 45°C. Twelve fractions were collected and vacuum-dried for further MS analysis, respectively.

#### Low pH Nano-HPLC-MS/MS analysis

Each fraction was re-suspended in 40μL solvent C (C: water with 0.1% formic acid; D: ACN with 0.1% formic acid), separated by nanoLC and analyzed by on-line electrospray tandem mass spectrometry, which was performed on an EASY-nLC 1000 system (Thermo Fisher Scientific, Waltham, MA) connected to an Orbitrap Fusion mass spectrometer (Thermo Fisher Scientific, San Jose, CA) equipped with an online nano-electrospray ion source. 4 μL peptide sample was loaded onto the analytical column (Acclaim PepMap C18, 75 μm x 25 cm), and eluted using a 110 min linear gradient from 5% D to 30% D at a flow rate of 300 nL/min. The Orbitrap Fusion mass spectrometer was operated in the data-dependent mode to switch automatically between MS and MS/MS acquisition. The Orbitrap Fusion mass spectrometer was operated in the data-dependent mode to switch automatically between MS and MS/MS acquisition. the scanning range of parent ion is 350–1600 m/z, the resolution of primary mass spectrometry is 60000 at 200 m/z, the AGC taget was set to 1000000, and the maximum injection time was 50 ms. The MS/MS acquisition was performed in Orbitrap with 3 s cycle time, the resolution was 15000 at m/z 200. Ions with charge states 2+, 3+ and 4+ were sequentially fragmented by higher energy collisional dissociation (HCD) with a normalized collision energy (NCE) of 30%. In all cases, one microscan was recorded using dynamic exclusion of 30 seconds.

### Data processing

The Chroma TOF 4.3X software of LECO Corporation combined with LECO-Fiehn Rtx5 database were employed to extract peak values and correct the original mass spectra data of metabonomics for principal component analysis (PCA) and partial least squares–discriminant analysis (PLS–DA). The standards were variable importance in the projection (VIP) >1.0 and T-test (P <0.05) to select differential metabolites [14].

Protein identifications were performed using the MASCOT search engine (version 2.3.2; Matrix Science, London, UK) embedded into Proteome Discoverer 1.4 (Thermo Electron, San Jose, CA, USA) [15] (Suplementary information). The LC/MS raw data were searched in the UniProt-reviewed rat protein database and differentially expressed proteins were screened by the criteria of up-regulation greater than 1.2 times or down-regulation less than 0.833 and T-test P value less than 0.05 [3]. The statistical analysis of related data was performed using SPSS software 20.0.

### Integrated metabonomics and proteomics analysis

To associate the proteomics data with the metabonomics data, we conducted a joint pathway enrichment analysis with differential metabolites and proteins in the KEGG database by Metaboanalyst platform, which entitles the weight strategy for different proteomics data universe and metabolomics data universe with a weighted z-test [16].

### Protein-protein interactions analysis

After identification of the key metabolic pathways based on differential metabolites and proteins, differential proteins involved in significantly perturbed joint pathways were further obtained for interaction analysis. Then, they all were imported into STRING database for protein-protein interactions analysis to link their interactive actions [17]. Further to search the hub proteins in the protein-protein interaction (PPI) network, topological parameters were calculated by four topological methods, including maximal clique centrality (MCC), density of maximum neighborhood component (DMNC), maximum neighborhood component (MNC), and Degree algorithms embedded in Cytoscape 3.8.2 [18].

## Results

### Results of metabolomics analysis

The GC-TOF/MS analysis was performed to detect metabolic profiles of liver tissues of rats in normal control group and MetS model group by using an Agilent 7890 gas chromatograph system combined with a time-of-flight mass spectrometer. In total, 696 ion peaks were identified out in two groups. Followed by searching the LECO/Fiehn metabolomics library, the majority of the peaks were identified as endogenous metabolites, and some of these peaks were attributed to the derivatives of byproducts. Besides, metabolic features detected in less than 50% of QC samples were removed. Finally, a total of 258 metabolites in the liver samples of two groups were matched and quantified.

**Fig 1:**
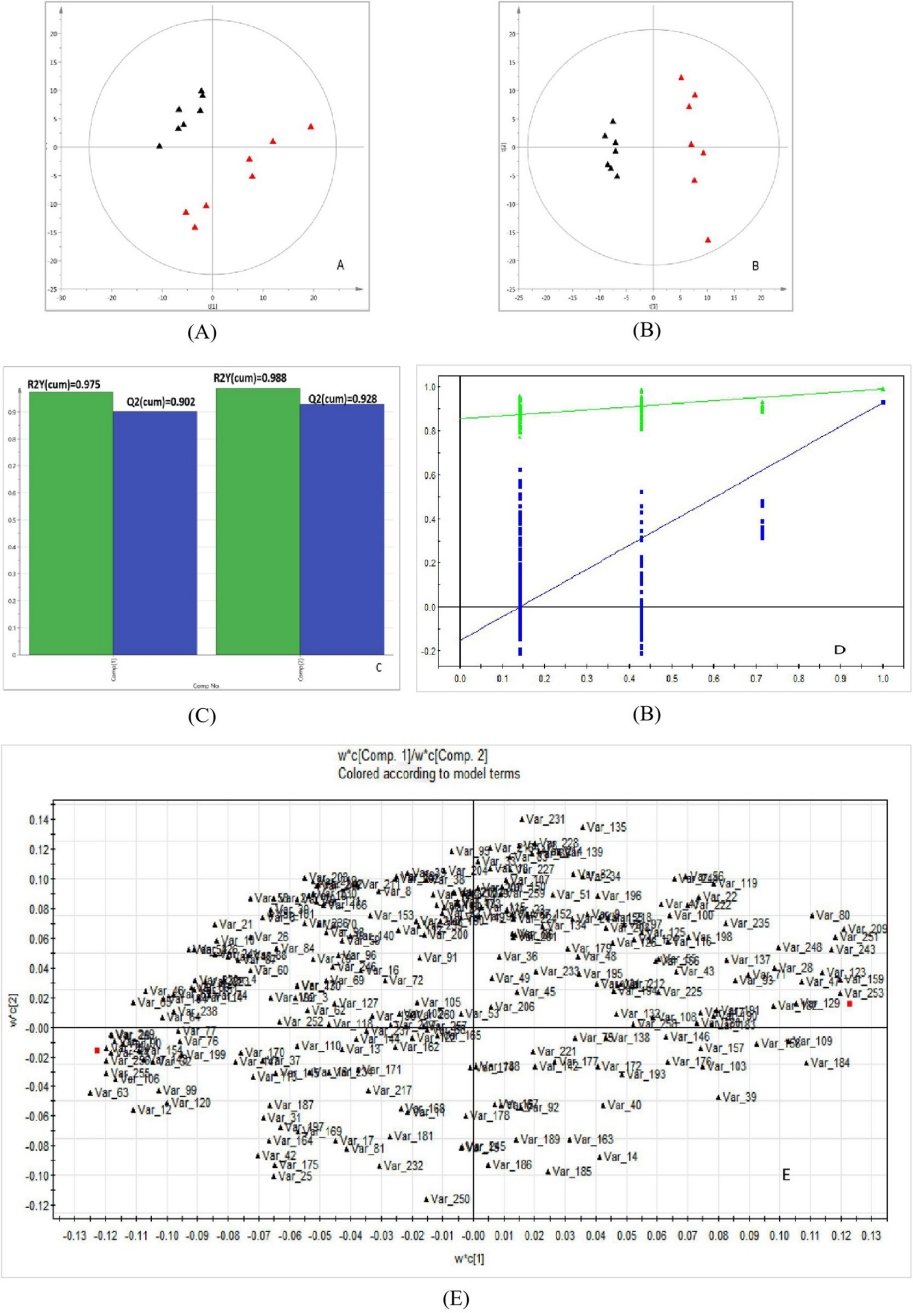
The metabolic profiles analysis of liver samples between two groups. (A) PCA score plots. (B) PLS–DA models. (C) Fit resutls of PLS-DA model. (D) permutation tests. Red represents the MetS model group, black represents the control group, Green △ is for R2Y (cum), and blue □ is for Q2Y (cum). (E)The loading score plot of PLS-DA model.

Then, pattern recognition techniques including PCA and PLS-DA were used to detect metabolic differences between two groups. PCA Fig 1(A) and PLS–DA (Fig 1(B)) were built by using SIMCA software (version 14.0, Sweden). The PCA result showed that some significant differences exist in the metabolic profiles of the samples but could not be clearly distinguished between groups. So, the supervised PLS-DA was performed to highlight the differences between groups and identify differential metabolites. The PLS-DA result showed that it can identify differential metabolites between two groups. The values of corresponding R2Y (cum) and leave-one-out cross-validation Q2Y (cum) values of PLS-DA model for the control and model groups were 0.988 and 0.928, as shown in Fig 1(C), showing that the derived model produced high predictive abilities and satisfactory effectiveness achieving distinct separations between the two comparison groups. Additionally, the 200 permutation tests (Fig 1(D)) were performed, and the results showed that the PLS–DA model was not overfitting and had high reliability. Therefore, the derived PLS-DA model can distinguish two groups. And, the loading plot of PLS-DA was presented in Fig 1(E).

In total, 82 significantly changed metabolites (VIP >1 and P <0.05) in liver tissues of rats were identified in two groups, which were accountable for momentous separations for PLS-DA model (S1 Table in S1 File). Among these metabolites, 50 metabolites exhibited higher abudance in the MetS group than those in the control group (P <0.05), including 2-ketobutyric acid, malonic acid, valine, isoleucine, proline, serine, threonine, methionine, phenylalanine, linolenic acid, tartronic acid, pyruvic acid, oxalic acid, hypoxanthine, pyruvate, allose and so on. While, the abudance of 32 metabolites in the MetS group were lower than that in the control group (P <0.05), including xylose, fructose, mannose, oleic acid, N-methyl-DL-alanine, oxoproline, 4-cholesten-3-one, phosphate, palatinose, etc.

### Proteomic analysis

**Fig 2:**
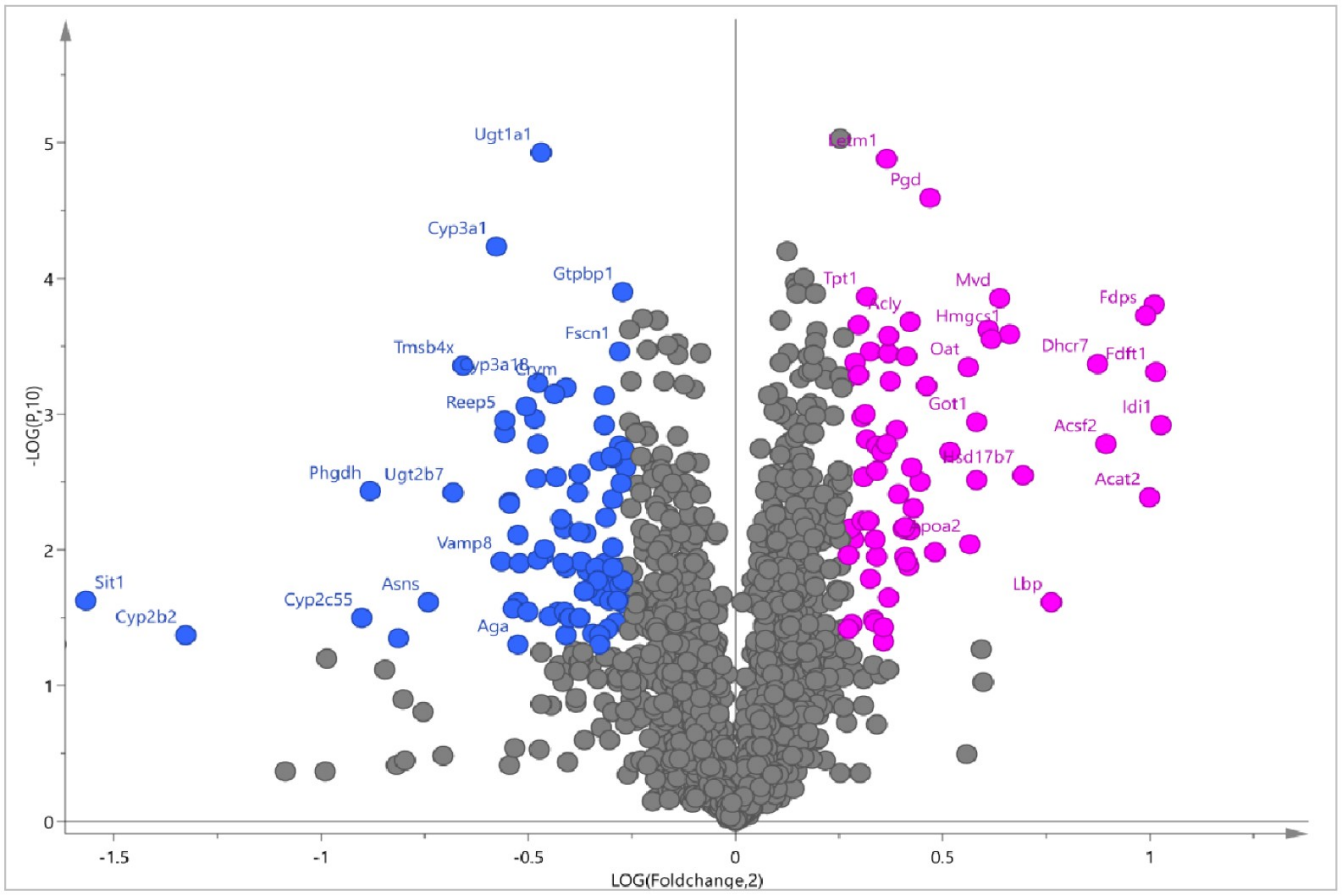
Differential proteins in liver tissues of rats between normal control and MetS groups. Rose red circle represents up-regulated protein and blue circle represents down-regulated protein.

The iTRAQ method was used for the proteomic analysis of liver tissues of rats in normal control group and MetS model group. By utilization of iTRAQ-based quantitative proteomics, we identified 2049 proteins in liver tissue samples of the two groups, among which 153 proteins exhibited significantly altered abundance (fold change >1.2 or <0.833, P >0.05) between two groups (S2 Table in S1 File). As shown in Fig 2, the abudance of 67 proteins was significantly increased and 86 proteins were significantly decreased in MetS group, which can be considered as differential proteins on the bases of coverage. The proteins with increased abudance mainly included: mitochondrial proton/calcium exchanger protein, 6-phosphogluconate dehydrogenase, diphosphomevalonate decarboxylase, “Acetyl-CoA acetyltransferase, cytosolic”, squalene synthase, ATP-citrate synthase, “aspartate aminotransferase, cytoplasmic”, “hydroxymethylglutaryl-CoA synthase, cytoplasmic”, farnesyl pyrophosphate synthase, isopentenyl-diphosphate delta-isomerase 1, etc. The proteins with decreased abudance mainly included: UDP glucuronosyltransferase family 1 member A1, GTP binding protein 1, “thymosin beta 4, X-linked”, signaling threshold regulating transmembrane adaptor 1, “cytochrome P450, family 2, subfamily b, polypeptide 2”, prostaglandin G/H synthase 1, cytochrome P450 3A18, signaling threshold-regulating transmembrane adapter 1, cholesterol 7-alpha-monooxygenase, acyl-coenzyme A thioesterase 12.

### Integrated proteomics and metabolomics analysis

To associate the proteomics data with the metabolomics data, we conducted a joint pathway enrichment analysis with differential metabolites and proteins in the KEGG database. As given in Table 1, 16 pathways were significantly enriched (P <0.05). By analyzing hit metabolites and proteins involved in these enriched pathways, 18 differential metabolites and 36 differential proteins were obtained. Tables 2 and 3 listed the information of these crucial differential metabolites and proteins, respectively. As shown in Table 1, the integrated analysis of differential proteins and metabolites revealed that 16 metabolic pathways were significantly perturbed (P <0.05), including retinol metabolism, alanine, aspartate and glutamate metabolism, arginine biosynthesis, valine, leucine and isoleucine biosynthesis, glycine, serine and threonine metabolism, arginine and proline metabolism, ascorbate and aldarate metabolism, cysteine and methionine metabolism, propanoate metabolism, terpenoid backbone biosynthesis, citrate cycle (TCA cycle), linoleic acid metabolism, pyruvate metabolism, pentose and glucuronate interconversions, steroid hormone biosynthesis, pantothenate and CoA biosynthesis. These metabolic pathways can be grouped into amino acid metabolism, fatty acid metabolism, glucose metabolism and biosynthetic pathways.

**Table 1:** Significantly perturbed metabolic pathways identified by integrated proteomics and metabolomics analysis.

Metabolic Pathways	Total	Hits	p-value	Impact
Retinol metabolism	45	10	5.63E-06	0.54545
Alanine, aspartate and glutamate metabolism	59	10	7.02E-05	0.51724
Arginine biosynthesis	27	6	0.00048	0.5
Valine, leucine and isoleucine biosynthesis	12	4	0.000875	0.36364
Ascorbate and aldarate metabolism	17	4	0.003613	0.1875
Glycine, serine and threonine metabolism	72	8	0.006405	0.47887
Propanoate metabolism	48	6	0.010217	0.3617
Arginine and proline metabolism	78	8	0.010336	0.44156
Terpenoid backbone biosynthesis	36	5	0.012165	0.54286
Cysteine and methionine metabolism	71	7	0.019849	0.64286
Citrate cycle (TCA cycle)	42	5	0.022807	0.43902
Linoleic acid metabolism	17	3	0.026903	0.5
Pyruvate metabolism	45	5	0.02987	0.45455
Pentose and glucuronate interconversions	32	4	0.034805	0.22581
Steroid hormone biosynthesis	175	12	0.038768	0.22989
Pantothenate and CoA biosynthesis	34	4	0.042299	0.30303

The “Total” column represents total numbers of metabolites and proteins involved in the pathways; The “Hits” column represents hit numbers of metabolites or proteins involved in the pathways.

**Table 2:** Differential metabolites involved in significantly perturbed metabolic pathways.

KEGG ID	Name	Fold change	p-value
C00022	Pyruvic Acid	1.609	0.0154
C00109	2-Ketobutyric Acid	1.165	0.0429
C05984	2-Hydroxybutyric Acid	2.315	0.0001
C00099	Beta-Alanine	2.342	0.0035
C00183	Valine	1.570	0.0000
C00407	Isoleucine	1.818	0.0000
C00148	Proline	1.581	0.0001
C00122	Fumaric Acid	0.785	0.0409
C00188	Threonine	1.201	0.0030
C00073	Methionine	1.389	0.0000
C00300	Creatine	1.740	0.0376
C00181	Xylose	0.247	0.0170
C00077	Ornithine	1.320	0.0145
C00864	Pantothenic Acid	2.038	0.0229
C00137	Myo-Inositol	0.225	0.0001
C05285	Adrenosterone	2.136	0.0162
C03681	5-Alpha-Dihydroprogesterone	0.460	0.0000
C05485	21-Hydroxypregnenolone	0.113	0.0036

**Table 3:** Differential proteins involved in significantly perturbed metabolic pathways.

Symbol	Uniprot	Protein name	Biological function	Fold	p-value
Abat	P50554	4-aminobutyrate aminotransferase, mitochondrial	Neurotransmitter degradation	1.237	0.0029
Acat2	Q5XI22	Acetyl-CoA acetyltransferase, cytosolic	Cholesterol biosynthesis	1.999	0.0041
Acly	P16638	ATP-citrate synthase	Lipid metabolism	1.338	0.0002
Acot12	Q99NB7	Acyl-coenzyme A thioesterase 12	Lipid metabolism	0.832	0.0019
Agxt	P09139	Serine–pyruvate aminotransferase, mitochondrial	Gluconeogenesis	1.236	0.0061
Aldh1a1	P51647	Retinal dehydrogenase 1	Retinoic acid biosynthesis	0.715	0.0011
Aldh4a1	P0C2X9	Delta-1-pyrroline-5-carboxylate dehydrogenase	Proline metabolism	1.222	0.0004
Aox3	Q5QE80	Aldehyde oxidase 3	Oxidoreductase	1.253	0.0003
Asns	P49088	Asparagine synthetase [glutamine-hydrolyzing]	Amino-acid biosynthesis	0.597	0.0245
Ass1	P09034	Argininosuccinate synthase	Amino-acid biosynthesis	1.378	0.0006
Bhmt	O09171	Betaine–homocysteine S-methyltransferase 1	Homocysteine metabolism.	1.314	0.0039
Cps1	P07756	Carbamoyl-phosphate synthase [ammonia]	Urea cycle	1.29	0.0003
Cth	P18757	Cystathionine gamma-lyase	Cysteine biosynthesis	1.277	0.0019
Cyp2b2	P04167	Cytochrome P450 2B2	Oxidoreductase	0.398	0.0421
Cyp2c7	P05179	Cytochrome P450 2C7	Oxidoreductase	1.332	0.0122
Cyp3a18	Q64581	Cytochrome P450 3A18	Oxidoreductase	0.719	0.0006
Cyp3a2	P05183	Cytochrome P450 3A2	Oxidoreductase	0.717	0.0029
Cyp7a1	P18125	Cholesterol 7-alpha-monooxygenase	Cholesterol metabolism	0.777	0.0200
Echdc1	Q6AYG5	Ethylmalonyl-CoA decarboxylase	Metabolite proofreading	1.433	0.0019
Enpp3	P97675	Ectonucleotide pyrophosphatase/phosphodiesterase 3	Hydrolase	0.803	0.0135
Fdps	P05369	Farnesyl pyrophosphate synthase	Isoprene biosynthesis	1.988	0.0002
Gls2	P28492	Glutaminase liver isoform, mitochondrial	Glutamine catabolism	1.527	0.0002
Got1	P13221	Aspartate aminotransferase, cytoplasmic	Amino-acid biosynthesis	1.497	0.0011
Hmgcs1	P17425	Hydroxymethylglutaryl-CoA synthase, cytoplasmic	Cholesterol biosynthesis	1.581	0.0003
Hsd17b7	Q62904	3-keto-steroid reductase	Lipid biosynthesis	1.618	0.0028
Idi1	O35760	Isopentenyl-diphosphate Delta-isomerase 1	Cholesterol biosynthesis	2.036	0.0012
Mvd	Q62967	Diphosphomevalonate decarboxylase	Cholesterol biosynthesis	1.555	0.0001
Oat	P04182	Ornithine aminotransferase, mitochondrial	Amino-acid biosynthesis.	1.475	0.0005
Pck1	P07379	Phosphoenolpyruvate carboxykinase, cytosolic [GTP]	Gluconeogenesis	1.248	0.0062
Phgdh	O08651	D-3-phosphoglycerate dehydrogenase	Amino-acid biosynthesis	0.542	0.0037
Pycr3	Q5PQJ6	Pyrroline-5-carboxylate reductase 3	Amino-acid biosynthesis	0.77	0.0028
Retsat	Q8VHE9	All-trans-retinol 13,14-reductase	Retinol metabolic process	0.832	0.0024
Sdhd	Q6PCT8	Succinate dehydrogenase [ubiquinone]	Carbohydrate metabolism	0.797	0.0424
Ugt1a1	Q64550	UDP-glucuronosyltransferase 1–1	Glycosyltransferase	0.722	0
Ugt1a5	Q64638	UDP-glucuronosyltransferase 1–5	Glycosyltransferase	0.694	0.0246
Ugt2b7	Q62789	UDP-glucuronosyltransferase 2B7	Lipid metabolism	0.623	0.0038

As shown in Table 1, all impact values of these pathways were greater than 0.2. Generally, an impact value equal to or greater than 0.1 indicates that this altered pathway is obviously affected. Thereby, these 16 enriched pathways were significantly perturbed in MetS model group. Thus, differential metabolites and proteins involved in these 16 perturbed pathways of MetS can be considered as significantly discriminant metabolites and proteins between two groups.

### PPI network construction

**Fig 3:**
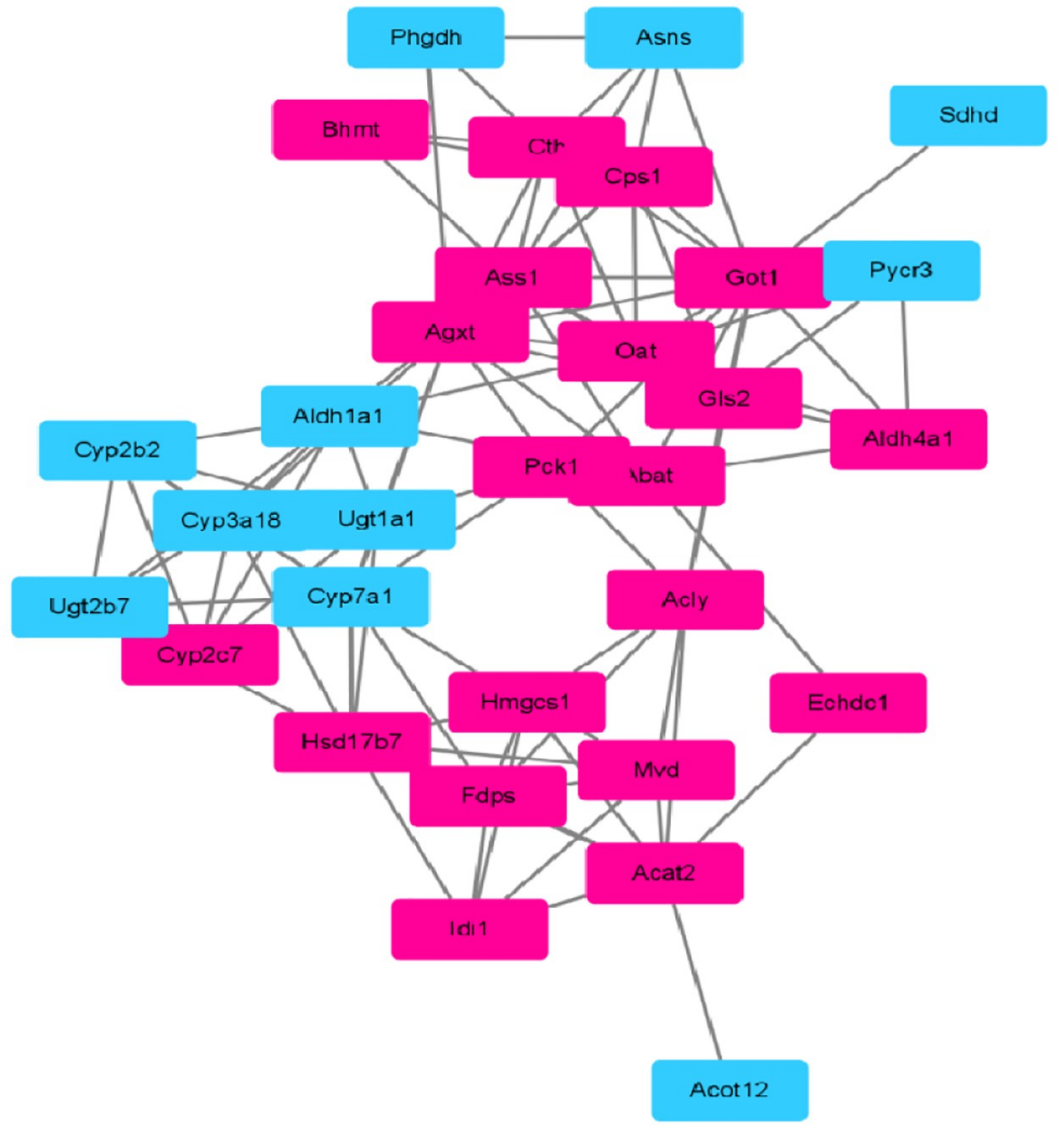
Protein-protein interactions network analysis. Rose red rectangle represents increased protein and blue rectangle represents decreased protein.

The 36 differential proteins were obtained after the joint pathway enrichment analysis with differential metabolites and proteins in the KEGG database (Table 3). To identify the hub proteins that have many interaction partners, protein-protein interactions analysis was first constructed based on string database by using Cytoscape 3.8.2 [18]. As a result, a total of 33 nodes and 98 edges were obtained from 36 genes with a PPI enrichment P-value smaller than 1.0e-16. As shown in Fig 3, 31 differential proteins interacted with each other, including 20 increased proteins and 11 decreased proteins in liver tissues of MetS group compared with the control group. Then, we used four calculation methods of topological parameters by cytoHubba plugin embedded in Cytoscape 3.8.2 to search hub proteins in the PPI network, including maximal clique centrality (MCC), density of maximum neighborhood component (DMNC), maximum neighborhood component (MNC), and Degree algorithm. The shared sets of proteins among these four topological methods were considered as hub proteins of the PPI network of MetS. The results showed that 16 of the top 20 hub proteins were shared among four methods, including Hmgcs1, Fdps, Acat2, Mvd, Hsd17b7, Cyp3a18, Cyp2c7, Cyp2b2, Cyp7a1, Ugt1a1, Got1, Ugt2b7, Aldh1a1, Ass1, Cps1 and Cth. Table 4 gave the topological parameters of these hub proteins in the PPI network. Among 16 hub proteins, 10 proteins were up-regulated in liver tissues of MetS group, and 6 proteins were down-regulated.

**Table 4:** The topological parameters of hub proteins in the interactive network of differential proteins.

Symbol	Protein name	Variationtype	MCC	DMNC	MNC	Degree
Acat2	Acetyl-CoA acetyltransferase, cytosolic	up	146	0.61814	6	8
Aldh1a1	Retinal dehydrogenase 1	down	52	0.58344	5	8
Ass1	Argininosuccinate synthase	up	40	0.358	9	9
Cps1	Carbamoyl-phosphate synthase [ammonia], mitochondrial	up	32	0.40246	7	7
Cth	Cystathionine gamma-lyase	up	24	0.36588	7	7
Cyp2b2	Cytochrome P450 2B2	down	96	0.61814	6	6
Cyp2c7	Cytochrome P450 2C7	up	96	0.61814	6	6
Cyp3a18	Cytochrome P450 3A18	down	114	0.52483	8	8
Cyp7a1	Cholesterol 7-alpha-monooxygenase	down	78	0.3791	10	10
Fdps	Farnesyl pyrophosphate synthase	up	150	0.54881	7	7
Got1	Aspartate aminotransferase, cytoplasmic	up	65	0.3733	11	12
Hmgcs1	Hydroxymethylglutaryl-CoA synthase, cytoplasmic	up	150	0.54881	7	7
Hsd17b7	3-keto-steroid reductase	up	138	0.40573	9	9
Mvd	Diphosphomevalonate decarboxylase	up	144	0.61814	6	6
Ugt1a1	UDP-glucuronosyltransferase 1–1	down	66	0.43736	8	8
Ugt2b7	UDP-glucuronosyltransferase 2B7	down	54	0.52304	6	6

## Discussion

Metabolic syndrome is a symptom group caused by abnormal metabolism of the three major nutrients of carbohydrates, lipids and proteins. The liver is the main organ of metabolism in the body and the center of the metabolism of various substances, which plays an important role in the metabolism of the three major nutrients. Therefore, changes in metabolites and proteins of liver tissues may be used to reveal possible mechanisms of MetS, which may also find the corresponding drug targets. The current research on the mechanism of metabolic syndrome mainly have been focused on the analysis of metabolites in the serum or urine samples. In this study, the differential metabolites and proteins of liver tissues of MetS rats were detected to explore possible mechanism of MetS, which may provide some new clues.

Through the fusion of liver metabolome and proteome data, 16 significantly perturbed pathways were obtained, including 6 pathways of amino acid metabolism, 2 pathways of lipid metabolism, 1 pathway of glucose metabolism and 7 pathways of biosynthetic pathways. Li et al. reported 15 metabolic pathways disrupted in MetS patients from serum metabolomics and 5 pathways were consistent with our finding based on liver tissues, including alanine, aspartate and glutamate metabolism, valine, leucine and isoleucine biosynthesis, glycine, serine and threonine metabolism, propanoate metabolism, and citrate cycle (TCA cycle) [19]. Sun et al. identified 17 metabolic pathways perturbed in patients with T2DM and 5 pathways were the same with our study, including alanine, aspartate and glutamate metabolism, valine, leucine and isoleucine biosynthesis, glycine, serine and threonine metabolism, arginine and proline metabolism, cysteine and methionine metabolism [20]. Among these perturbed pathways, three pathways of amino acid metabolisms were shared by patients with MetS and diabetes and our study, including alanine, aspartate and glutamate metabolism, valine, leucine and isoleucine biosynthesis, and glycine, serine and threonine metabolism. Additionally, recent reports found that aberrant levels of glycine and serine caused by glycine and serine and threonine dysmetabolism were associated with obesity, insulin resistance and metabolic syndrome [21]. Valine, leucine and isoleucine belong to the branch-chain amino acids, which were repeatedly confirmed to be altered in T2DM and MetS patients attributed to their activity of insulin-induced impairment [22].

As shown in Table 1, our result also revealed that the TCA cycle was significantly perturbed in liver tissues of MetS rats caused by differential levels of pyruvic acid, fumaric acid, Acly, Sdhd and Pck1. The TCA cycle is the final metabolic pathway of carbohydrates, lipids and amino acids, and is also the hub of the metabolic links among them, which was confirmedly associated with metabolic diseases such as T2DM. The Acly enzyme cleaves cytosolic citrate to produce acetyl-CoA, and is up-regulated after consumption of carbohydrates, which plays a crucial role in lipid metabolisms. Currently, inhibition of Acly as a treatment for metabolic diseases is being tried in clinical trials [23]. Pck1 is an important rate-limiting enzyme in gluconeogenesis, which shows a significant role in gluconeogenesis. Rodent models also demonstrated that over-expression of Pck1 can result in T2DM development [24].

Additionally, as shown in Table 2, 18 differential metabolites were obtained, which were involved in 16 significantly perturbed metabolic pathways. These 18 differential metabolites can be considered as significantly discriminant metabolites in liver tissues between the MetS and control groups, including pyruvic acid, 2-ketobutyric acid, 2-hydroxybutyric acid, alanine, valine, isoleucine, proline, fumaric acid, threonine, methionine, creatine, xylose, ornithine, pantothenic acid, myo-inositol, adrenosterone, 5-alpha-dihydroprogesterone, 21-hydroxypregnenolone. Among these metabolites, the level of alanine was the most changed. Alanine is vital in the glucose-alanine cycle in mammalian liver tissues. Numerous studies have shown that alterations to the alanine cycle, leading to increased levels of ALT (Alanine aminotransferase) may have implications in the development of T2DM and hyperglycemia [6]. Furthermore, alanine levels were increased in obesity and correlate with visceral adiposity in a Japanese population [25]. The difference in concentrations of valine and isoleucine was greatest between the two groups. Valine and isoleucine belong to the branch-chain amino acids, which were reported to be altered in T2DM and MetS patients attributed to their activity of insulin-induced impairment [26].

As listed in Table 3, a total of 36 differential proteins were also involved in these significantly perturbed joint pathways. These differential proteins belong to cytochrome P450 family, HMG-CoA reductase family, the UDP-glycosyltransferase family, rgininosuccinate synthase family, 6-phosphogluconate dehydrogenase family, the phosphoenolpyruvate carboxykinase [GTP] family, which referred to metabolic processes of gamma-aminobutyric acid, pyrimidine-containing compound and acetyl-CoA, cellular response to glucagon stimulus, biosynthetic processes of cholesterol and amino acids.

Based on the results of PPI network analysis and topological parameter calculation of four methods, 16 proteins were considered as hub proteins of the PPI network of MetS, including 10 up-regulated proteins (Hmgcs1, Fdps, Acat2, Mvd, Hsd17b7, Cyp2c7, Got1, Ass1, Cps1, Cth) and 6 down-regulated proteins (Cyp3a18, Cyp2b2, Cyp7a1, Ugt1a1, Ugt2b7, Aldh1a1). Significantly, 5 of the 16 hub proteins were engaged in amino acid biosynthetic processes, including Aldh1a1, Ass1, Cps1, Cth and Got1. Additionally, 7 hub proteins including Cyp7a1, Acat2, Fdps, Got1, Hmgcs1, Hsd17b7 and Mvd were engaged in cholesterol biosynthetic processes. Besides, Aldh1a1, Fdps, Hmgcs1, Mvd were involved in isoprenoid biosynthetic processes. Among the 16 hub proteins, the degrees of Cyp7a1 and Got1 in the network ranked top two. Generally, the greater a node’s degree is, the more significant the node is in the interactive network [27]. Thus, Cyp7a1 and Got1 may be more important in the PPT network. CYP7a1 is a rate-limiting enzyme that catalyzes the decomposition of cholesterol into bile acids in the liver, and maintains the balance of cholesterol metabolism. Here, the protein abudance of CYP7a1 was decreased in MetS group, which was also found the same trend in type 2 diabetes rats [28]. This may be related with that high fat diet can cause repressed mRNA level and protein level of Cyp7A1 in rats [29]. Got1 (Ast1) is one of the important transaminase enzymes, which is an indicator of liver function tests to determine whether the liver is damaged in clinic practices. Here, the increased abudance of Got1 in MetS group implied abnormal liver function in MetS. This was consistent with the report that serum level of Got (Ast) can be used as a predictor of MetS based on the MetS incidence of 4053 individuals in 4-year continuous physical examination [30]. Therefore, Cyp7a1 and Got1 may be potential therapeutic targets of MetS.

Through protein and drug query in the PubMed database, it is found that both atorvastatin and resveratrol can increase the expression of Cyp7a1 [31, 32] and decrease the expression of Got1 [33, 34], which regulate the cholesterol level in the blood, and inhibit the formation of blood clots from platelets to adhere to the blood vessel wall, thereby inhibitting and reducing the occurrence and development of cardiovascular diseases. Thus, these two drugs may be potential drugs for the treatment of MetS. Therefore, molecular docking was carry out to understanding their interaction with two proteins.

The docking simulation was carried out using the MOE2008 by following steps. First, the three dimension crystal structures of Cyp7a1 complex with cholest-4-en-3-one (PDB code: 3sn5) and Got1 complex with pyridoxal-5’-phosphate (PDB code: 3ii0) were retrieved from the RSCB protein databank. Then, the proteins were protonated using AMBER99 force field and minimized with a RMSD (Root Mean Squared Deviation) gradient of 0.05 kcal/mol Å. Additionally, the ligand atom mode was utilized to define the binding site, and the docking placement was using triangle matcher algorithm. Finally, two rescoring methods including London dG and Affinity dG, together with a force field were adopted to compute the interactions.

**Fig 4:**
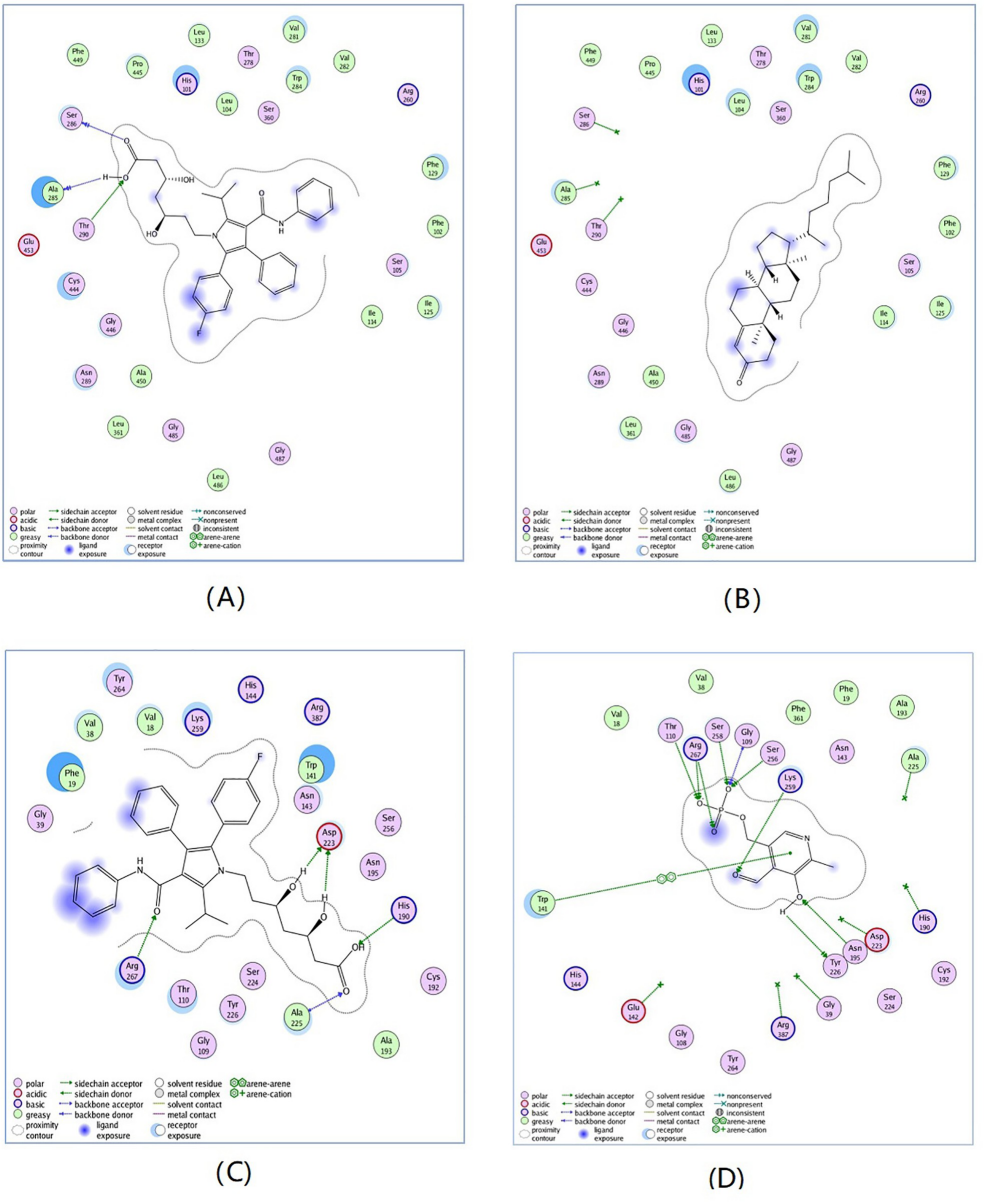
Comparison between the binding modes of atorvastatin. (A) and cholest-4-en-3-one; (B) in the Cyp7a1 active site and Atorvastatin; (C) and pyridoxal-5’-phosphate; (D) in the Got1 active site.

As shown in Fig 4, atorvastatin can well bind to Cyp7a1 and Got1, and the binding affinity was greater than that of two enzyme substrates (cholest-4-en-3-one and pyridoxal-5’-phosphate). This may be caused by the molecular structures of atorvastatin, of which the conformation was in good agreement with the amino acid residues of the active sites of enzymes, and easily formed stronger interactions such as hydrophobic action and hydrogen bonding than two enzyme substrates. These docking results further confirmed that atorvastatin can increase activity of Cyp7a1 and inhibit activity of Got1. Although, resveratrol can bind to Cyp7a1 and Got1 with good docking scores, the binding affinity was not better than that of two enzyme substrates, which may be due to its relatively rigid skeleton. Additionally, these two drugs have been also investigated for MetS in many clinical and animal studies [35–37]. Therefore, it can be confirmed that atorvastatin and resveratrol may be possible drugs for prevention and treatment for MetS.

## Conclusion

Currently, the combination of multiple omics has become a hot spot in the study of disease mechanisms. To the best of our knowledge, we reported for the first time an integrated analysis of proteomic and metabolic profiles in liver tissues to reveal possible mechanisms of MetS, which can reflect molecular processes closer to the disease state than serum samples. In the metabonomic analysis, 82 differential metabolites were detected, of which the abudance of 50 metabolites were significantly increased and those of 32 metabolites were significantly decreased in liver tissues of MetS group. Additionally, a total of 153 proteins with significant changes were identified by quantitative proteomic analysis, of which the abudance of 67 proteins were significantly increased and those of 86 proteins were significantly decreased in liver tissues of MetS rats. After the joint pathway enrichment analysis of differential proteins and metabolites, 16 significantly perturbed pathways were identified (P <0.05), of which 3 pathways were shared by patients with metabolic syndrome and diabetes identified by serum samples, including alanine, aspartate and glutamate metabolism, valine, leucine and isoleucine biosynthesis, and glycine, serine and threonine metabolism. Additionally, it was found that 18 differential metabolites were closely related with 36 differential proteins, which can be considered as significantly discriminant metabolites and proteins in liver tissues between two groups, which referred to metabolic processes of gamma-aminobutyric acid, pyrimidine-containing compound and acetyl-CoA, cellular response to glucagon stimulus, biosynthetic processes of cholesterol and amino acids. Based on PPI network analysis and topological parameter calculation results of four methods, 16 differential proteins were considered as hub proteins of the PPI network of MetS, including 10 up-regulated proteins (Hmgcs1, Fdps, Acat2, Mvd, Hsd17b7, Cyp2c7, Got1, Ass1, Cps1, Cth) and 6 down-regulated proteins (Cyp3a18, Cyp2b2, Cyp7a1, Ugt1a1, Ugt2b7, Aldh1a1), mainly involved in metabolisms of amino acid and cholesterol, especially for Cyp7a1 and Got1 with top degree in PPI network, which may be considered as potential targets for prevention and treatment of MetS. Followed by searching the PubMed database and molecular docking of Cyp7a1 and Got1, it can be concluded that atorvastatin and resveratrol may be potential drugs for prevention and treatment for MetS, as both drugs have also been investigated for MetS in many clinical practices and animal models. In summary, our results revealed some candidates for proteins, pathways and drugs for the prevention and treatment of MetS, which may give some insights into the mechanism of MetS.

## Supporting information

S1 File (PDF)
