# Correction: Investigation into potential mechanisms of metabolic syndrome by integrative analysis of metabolomics and proteomics

**DOI:** 10.1371/journal.pone.0315213

**Published:** 2024-12-04

**Authors:** Meimei Chen, Zhaoyang Yang, Huijuan Gan, Yang Wang, Candong Li, Yuxing Gao

The third and fifth authors’ names are spelled incorrectly. The correct names are: Huijuan Gan and Candong Li. The correct citation is: Chen M, Yang Z, Gan H, Wang Y, Li C, Gao Y (2022) Investigation into potential mechanisms of metabolic syndrome by integrative analysis of metabolomics and proteomics. PLOS ONE 17(7): e0270593. https://doi.org/10.1371/journal.pone.0270593

## References

[1] Chen M, Yang Z, Gan H, Wang Y, Li C, Gao Y (2022) Investigation into potential mechanisms of metabolic syndrome by integrative analysis of metabolomics and proteomics. PLOS ONE 17(7): e0270593. doi: 10.1371/journal.pone.0270593 35789338 PMC9255766

