# Supplementary material for: Investigation into potential mechanisms of metabolic syndrome by integrative analysis of metabolomics and proteomics
Source: PLoS One. 2022 Jul 5;17(7):e0270593. doi: 10.1371/journal.pone.0270593 (PMC9255766; doi:10.1371/journal.pone.0270593)
Supplement: S1 File — (PDF) [file pone.0270593.s001.pdf]

Supplementary information

1. Animal modeling method [1]

All adult male Wistar rats (2 months) weighing 200±20g were housed in a 12/12 h light/dark cycle at an ambient temperature of 20 ± 2 °C and relative humidity of 60% - 65%. The normal control group were fed with normal diet. The MetS model group were fed with high-sugar-fat-diet (50% of normal diet, 18% sugar, 10% lard, 12% egg yolk powder, 2% cholesterol, 7.5% salt and 0.5% bile salt). After 15 weeks, MetS model group were fed with high-fat emulsion (ingredients: 10% of cholesterol, 30% of lard, 2% of sodium cholate, 0.5% of propylthiouracil, 10% of tween 80 and 10% of propylene glycol) by intragastric at a dose of 10 mL/kg/d for 2 weeks. The metabolic disorder of the animals was assessed by measuring abdominal perimeters, serum levels of HDL-C and insulin, and insulin-resistances (HOMA-IR, estimated using the homeostasis model assessment) according to the WHO definition [2]. After 17 weeks, all these parameters of MetS group were significantly different from that of normal control group, the specific result can be seen in Figure S1. Data were presented as the mean ± standard error. Statistical significance ($P < 0.05$) was determined using Two-sample T-test in SPSS software 20.0.

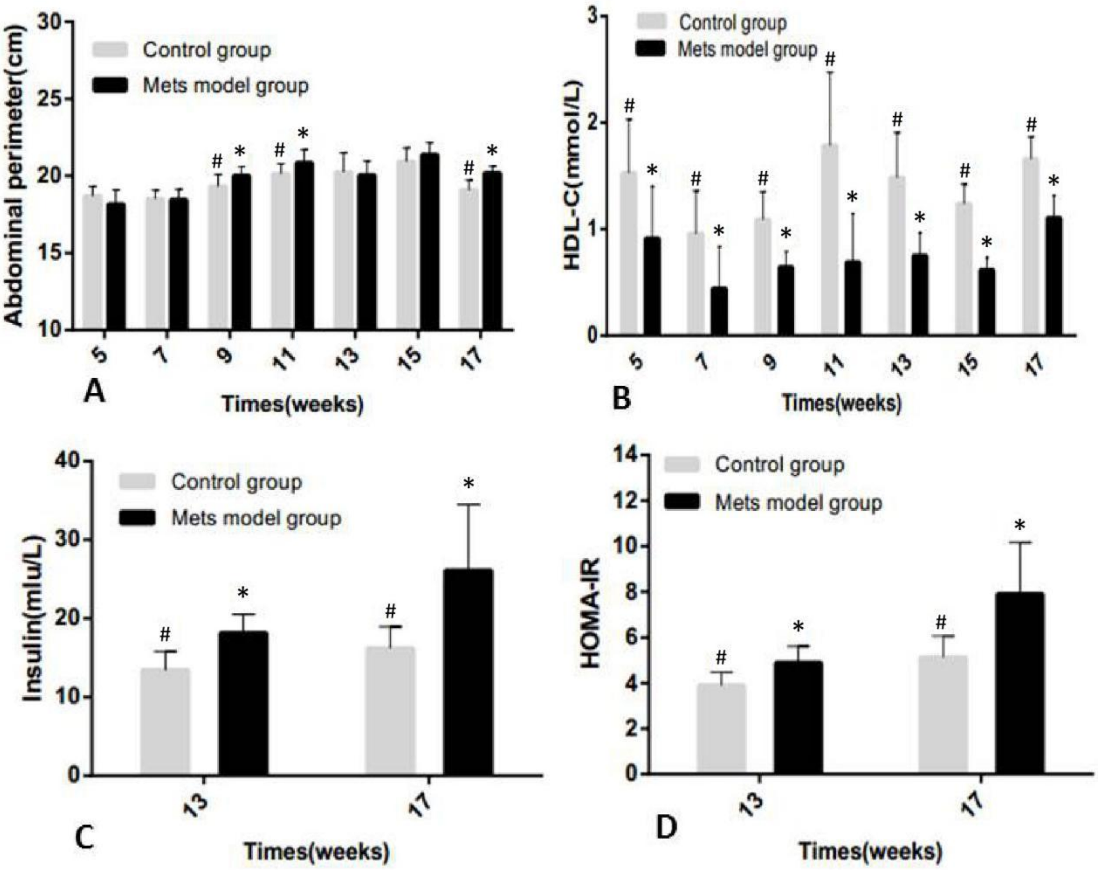

Figure S1. Biochemistry values of Wistar rats during 17 weeks diet feeding: (A) abdominal perimeter; (B) serum HDL level; (C) serum insulin level; (D) HOMA-IR . # $P < 0.05$ vs. Mets model group, * $P < 0.05$ vs. control group by t-test.

1. Z. Chen, H. Gan, J. Kang, Y. Guo, J. Zhang, M. Chen, Study on modeling method of sputum syndrome of metabolic syndrome in rats [J]. J. Liaoning Univ. Tradit. Chin. Med., 2017, 19(11):75-78.
2. WHO. Definition, diagnosis and classification of diabetes mellitus and its complications. diabetic medicine,

1999.

2. iTRAQ Labeling

After digested with sequence-grade modified trypsin (Promega, Madison, WI) at 37 ° C for 12 hours, the resultant peptide mixture was labeled using chemicals from the iTRAQ8Plex reagent kit (AB Sciex, CA, USA) according to the manufacturer's protocol. The MetS samples were labelled with isobaric tags 113, 114 and 115. The control samples were labelled with isobaric tags 116, 118 and 119. Internal standard (equivalent MetS and control) was labelled with isobaric tag 121.

3. Data Analysis

MS/MS spectra were searched using MASCOT engine (Matrix Science, London, UK; version 2.3.2) embedded into Proteome Discoverer 1.4. The following parameters were set.

**Item**	**Value**
Fixed modifications	Carbamidomethyl (C), iTRAQ 8plex（K），iTRAQ 8plex（N-term）
Variable modifications	Oxidation（M）； iTRAQ 8plex（Y）
Peptide Mass Tolerance	10 ppm
MS/MS Tolerance	50mmu
Max missed cleavages	2
Enzyme	Trypsin
Database	UniprotKB Swiss-Prot（Rattus norvegicus (Rat)）
Protein Quantification	The protein ratios are calculated as the median of only unique peptides of the protein
Experimental Bias	Normalizes all peptide ratios by the median protein ratio. The median protein ratio should be 1 after the normalization.

Table S1. Differential metabolites in liver tissue samples of rats between normal and MetS groups

**Metabolites**	**Abudance variation**	**Metabolites**	**Abudance variation**
2-Ketobutyric Acid	up	D-Glucoheptose	down
N-Methyl-Dl-Alanine	down	Oleic Acid	down
Malonic Acid	up	Linolenic Acid	up
3-Methylamino-1,2-Propanediol	down	Cis-Gondoic Acid	down
Valine	up	Xanthosine	up
Hydroxyurea	up	Guanosine	up
Guaiacol	up	Palatinose	down
Ethanolamine	up	5-Alpha-Dihydroprogesterone	down
Nicotinic Acid	up	Alpha-Tocopherol	up
Isoleucine	up	4-Cholesten-3-One	down
Proline	up	Pyruvic Acid	up
Maleic Acid	down	Oxalic Acid	up
Itaconic Acid	up	2-Hydroxybutanoic Acid	up

Fumaric Acid	down	Sulfuric Acid	up
Pyrrole-2-Carboxylic Acid	up	Phosphate	down
Tartronic Acid	up	Serine	up
4-Methyl-5-Thiazolethanol	up	Cycloleucine	up
Threonine	up	1-Indanol	up
Hydroquinone	down	Beta-Alanine	up
Thymine	up	3-Methylthiopropylamine	up
Biuret	up	Phosphoglycolic Acid	down
Maleamate	down	Threo-Beta-Hyrdoxyaspartate	up
Nicotinamide	up	Beta-Glutamic Acid	up
Threitol	up	5-Aminovaleric Acid	down
Methionine	up	Creatine	up
Oxoproline	down	Taurine	up
Phenylalanine	up	Flavin Adenine	down
Allose	up	Fructose	down
Pyrophosphate	down	Gluconic Lactone	down
Xylose	down	Mannose	down
Phthalic Acid	down	Guanidinosuccinic Acid	down
D-(Glycerol 1-Phosphate)	down	Myo-Inositol	down
Dehydroshikimic Acid	up	Cellobiose	down
Hypoxanthine	up	2-Monoolein	up
Ornithine	up	Prunin	up
1,5-Anhydroglucitol	up	Adrenosterone	up
Beta-Mannosylglycerate	up	21-Hydroxypregnenolone	down
Pantothenic Acid	up	5-Dihydrocortisol	down
L-Dopa	down	Zymosterol	up
N,N-Dimethylarginine	down	Luteolin	up
Glucoheptonic Acid	down	Stigmasterol	up

Table S2. Differential proteins in liver tissue samples of rats between normal and MetS groups

Symbol	Protein name	Fold change	P-value
Dhcr7	7-Dehydrocholesterol Reductase	1.835	0.0004
Nap1l1	Nucleosome Assembly Protein 1-Like 1	0.805	0.0059
Fads2	Fatty Acid Desaturase 2	1.345	0.0049
Tpmt	Thiopurine S-Methyltransferase	1.217	0.0083
Mpeg1	Macrophage-Expressed Gene 1 Protein	0.802	0.0173
Vamp8	Vesicle-Associated Membrane Protein 8	0.675	0.0124
Epb41l1	Band 4.1-Like Protein 1	0.821	0.0210
Crym	Ketimine Reductase Mu-Crystallin	0.754	0.0006
Ccs	Copper Chaperone For Superoxide Dismutase	1.244	0.0015
Ebp	3-Beta-Hydroxysteroid-Delta(8),Delta(7)-Isomerase	1.280	0.0474
Hrg	Histidine-Rich Glycoprotein	0.788	0.0414

Acot12	Acyl-Coenzyme A Thioesterase 12	0.832	0.0019
Vmp1	Vacuole Membrane Protein 1	0.780	0.0142
Retsat	All-Trans-Retinol 13,14-Reductase	0.832	0.0024
Higd1a	Hig1 Domain Family Member 1A, Mitochondrial	0.819	0.0347
Dcps	m7Gpppx Diphosphatase	0.808	0.0387
Pgpep1	Pyroglutamyl-Peptidase 1	1.308	0.0013
Sdhd	Succinate Dehydrogenase [Ubiquinone] Cytochrome b Small Subunit, Mitochondrial	0.797	0.0424
Hsd17b11	Estradiol 17-Beta-Dehydrogenase 11	0.685	0.0044
Echdc1	Ethylmalonyl-Coa Decarboxylase	1.433	0.0019
Stn1	Cst Complex Subunit Stn1	0.743	0.0286
Hbs1l	Hbs1-Like Protein	0.797	0.0219
Pla2g15	Group Xv Phospholipase A2	0.778	0.0076
Cyp51a1	Lanosterol 14-Alpha Demethylase	2.016	0.0002
Ugt1a5	Udp-Glucuronosyltransferase 1-5	0.694	0.0246
Cyp3a18	Cytochrome P450 3A18	0.719	0.0006
Ugt1a1	Udp-Glucuronosyltransferase 1-1	0.722	0.0000
Ptgs1	Prostaglandin G/H Synthase 1	0.803	0.0012
Azgp1	Zinc-Alpha-2-Glycoprotein	0.824	0.0136
Gbp2	Guanylate-Binding Protein 1	0.753	0.0136
Lbp	Lipopolysaccharide-Binding Protein	1.695	0.0246
Mvd	Diphosphomevalonate Decarboxylase	1.555	0.0001
Hsd17b7	3-Keto-Steroid Reductase	1.618	0.0028
Ugt2b7	Udp-Glucuronosyltransferase 2B7	0.623	0.0038
Tomm20	Mitochondrial Import Receptor Subunit Tom20 Homolog	1.210	0.0070
Cd81	Cd81 Antigen	1.227	0.0002
Rap1b	Ras-Related Protein Rap-1b	1.266	0.0111
Gadd45gip1	Growth Arrest And Dna Damage-Inducible Proteins-Interacting Protein 1	1.329	0.0111
Letm1	Mitochondrial Proton/Calcium Exchanger Protein	1.288	0.0000
Ghitm	Growth Hormone-Inducible Transmembrane Protein	0.767	0.0038
Eepd1	Endonuclease/Exonuclease/Phosphatase Family Domain-Containing Protein 1	1.261	0.0328
Acat2	Acetyl-Coa Acetyltransferase, Cytosolic	1.999	0.0041
Tmem97	Sigma Intracellular Receptor 2	1.264	0.0341
Ryden	Repressor Of Yield Of Denv Protein Homolog	0.724	0.0111
Aox3	Aldehyde Oxidase 3	1.253	0.0003
Pycr3	Pyrroline-5-Carboxylate Reductase 3	0.770	0.0028
Rsrc1	Serine/Arginine-Related Protein 53	1.283	0.0377
Uqcrh	Cytochrome b-c1 Complex Subunit 6, Mitochondrial	1.338	0.0071
Fgl1	Fibrinogen-Like Protein 1	0.820	0.0190
Sit1	Signaling Threshold-Regulating Transmembrane Adapter 1	0.338	0.0236
Cnrip1	Cb1 Cannabinoid Receptor-Interacting Protein 1	0.732	0.0307
Cd302	Cd302 Antigen	0.830	0.0252

Stbd1	Starch-Binding Domain-Containing Protein 1	0.814	0.0021
Rab18	Ras-Related Protein Rab-18	0.738	0.0007
Slc25a18	Mitochondrial Glutamate Carrier 2	1.325	0.0069
Dhtkd1	Probable 2-Oxoglutarate Dehydrogenase E1 Component Dhktd1, Mitochondrial	1.268	0.0017
Serpinb1a	Leukocyte Elastase Inhibitor A	0.773	0.0122
Acsf2	Acyl-Coa Synthetase Family Member 2, Mitochondrial	1.860	0.0017
Anxa2	Annexin A2	0.781	0.0198
Fdft1	Squalene Synthase	2.018	0.0005
Enpp3	Ectonucleotide Pyrophosphatase/Phosphodiesterase Family Member 3	0.803	0.0135
Slc27a2	Very Long-Chain Acyl-Coa Synthetase	0.813	0.0043
Pgd	6-Phosphogluconate Dehydrogenase, Decarboxylating	1.384	0.0000
Fscn1	Fascin	0.824	0.0003
H3f3b	Histone H3.3	1.291	0.0225
Fabp4	Fatty Acid-Binding Protein, Adipocyte	0.750	0.0285
Mapk1	Mitogen-Activated Protein Kinase 1	0.797	0.0499
Tpt1	Translationally-Controlled Tumor Protein	1.246	0.0001
Cycs	Cytochrome c, Somatic	1.290	0.0004
Cnbp	Cellular Nucleic Acid-Binding Protein	1.214	0.0349
Tmsb4x	Thymosin Beta-4	0.635	0.0004
Pon1	Serum Paraoxonase/Arylesterase 1	0.802	0.0126
Aif1	Allograft Inflammatory Factor 1	0.696	0.0077
Pctp	Phosphatidylcholine Transfer Protein	1.208	0.0379
Aldh1a1	Retinal Dehydrogenase 1	0.715	0.0011
Abat	4-Aminobutyrate Aminotransferase, Mitochondrial	1.237	0.0029
S100a8	Protein S100-A8	0.792	0.0135
Ste2	Estrogen Sulfotransferase Ste2	1.327	0.0067
Asns	Asparagine Synthetase [Glutamine-Hydrolyzing]	0.597	0.0245
Lss	Lanosterol Synthase	1.335	0.0134
Nrdc	Nardilysin	1.205	0.0005
Sec11a	Signal Peptidase Complex Catalytic Subunit Sec11A	0.812	0.0177
Psmb2	Proteasome Subunit Beta Type-2	0.822	0.0137
Crip2	Cysteine-Rich Protein 2	0.823	0.0181
Sdc2	Syndecan-2	1.263	0.0084
Cyp2c55	Cytochrome P450 2C55	0.535	0.0319
Slc25a1	Tricarboxylate Transport Protein, Mitochondrial	1.331	0.0004
Vim	Vimentin	0.795	0.0166
Gls2	Glutaminase Liver Isoform, Mitochondrial	1.527	0.0002
Hprt1	Hypoxanthine-Guanine Phosphoribosyltransferase	0.809	0.0235
Ctsd	Cathepsin D	0.752	0.0069
Hal	Histidine Ammonia-Lyase	1.210	0.0110
Hpx	Hemopexin	0.823	0.0017
Cyp2c70	Cytochrome P450 2C70	0.690	0.0272

Cth	Cystathionine Gamma-Lyase	1.277	0.0019
Cyp7a1	Cholesterol 7-Alpha-Monooxygenase	0.777	0.0200
Hmgcs1	Hydroxymethylglutaryl-Coa Synthase, Cytoplasmic	1.581	0.0003
Acly	Atp-Citrate Synthase	1.338	0.0002
St2a1	Bile Salt Sulfotransferase	1.496	0.0031
Anpep	Aminopeptidase N	0.797	0.0022
Anxa3	Annexin A3	0.752	0.0430
Cp	Ceruloplasmin	0.826	0.0032
Got1	Aspartate Aminotransferase, Cytoplasmic	1.497	0.0011
Fasn	Fatty Acid Synthase	1.361	0.0031
Mt-atp8	Atp Synthase Protein 8	1.343	0.0024
Dbi	Acyl-Coa-Binding Protein	1.536	0.0003
Ces1c	Carboxylesterase 1C	0.803	0.0007
Cd74	H-2 Class Ii Histocompatibility Antigen Gamma Chain	0.696	0.0127
Aldh4a1	Delta-1-Pyrroline-5-Carboxylate Dehydrogenase, Mitochondrial	1.222	0.0004
Agxt	Serine--Pyruvate Aminotransferase, Mitochondrial	1.236	0.0061
Ass1	Argininosuccinate Synthase	1.378	0.0006
Kng1	Kininogen-1	0.718	0.0118
Lgals3	Galectin-3	0.570	0.0445
Cyp4a10	Cytochrome P450 4A10	0.718	0.0016
Cps1	Carbamoyl-Phosphate Synthase [Ammonia], Mitochondrial	1.290	0.0003
Ephx1	Epoxide Hydrolase 1	0.706	0.0009
Pck1	Phosphoenolpyruvate Carboxykinase, Cytosolic [Gtp]	1.248	0.0062
Atp1b1	Sodium/Potassium-Transporting Atpase Subunit Beta-1	1.234	0.0011
S100a6	Protein S100-A6	0.706	0.0282
S100a10	Protein S100-A10	0.746	0.0060
Mt-atp6	Atp Synthase Subunit a	0.685	0.0045
G6pdx	Glucose-6-Phosphate 1-Dehydrogenase	1.228	0.0005
Fdps	Farnesyl Pyrophosphate Synthase	1.988	0.0002
Cyp3a2	Cytochrome P450 3A2	0.717	0.0029
Cyp2c7	Cytochrome P450 2C7	1.332	0.0122
Rbp4	Retinol-Binding Protein 4	0.831	0.0018
Cyp3a1	Cytochrome P450 3A1	0.670	0.0001
Apoa2	Apolipoprotein A-Ii	1.481	0.0091
Oat	Ornithine Aminotransferase, Mitochondrial	1.475	0.0005
Cyp2b2	Cytochrome P450 2B2	0.398	0.0421
Gpx1	Glutathione Peroxidase 1	1.287	0.0016
Ttr	Transthyretin	0.681	0.0014
Orm1	Alpha-1-Acid Glycoprotein	0.771	0.0075
Fgg	Fibrinogen Gamma Chain	0.827	0.0169
Map1	T-Kininogen 1	0.742	0.0029
Por	Nadph--Cytochrome P450 Reductase	0.814	0.0095
Marc2	Mitochondrial Amidoxime Reducing Component 2	1.265	0.0026

Ggcx	Vitamin K-Dependent Gamma-Carboxylase	0.812	0.0021
Aspg	60 Kda Lysophospholipase	1.241	0.0010
Cacna1g	Voltage-Dependent T-Type Calcium Channel Subunit Alpha-1G	1.252	0.0165
Slco1a4	Solute Carrier Organic Anion Transporter Family Member 1A4	0.822	0.0237
Idi1	Isopentenyl-Diphosphate Delta-Isomerase 1	2.036	0.0012
Hsd3b7	3 Beta-Hydroxysteroid Dehydrogenase Type 7	0.757	0.0314
Bhmt	Betaine--Homocysteine S-Methyltransferase 1	1.314	0.0039
Phgdh	D-3-Phosphoglycerate Dehydrogenase	0.542	0.0037
Rnf123	E3 Ubiquitin-Protein Ligase Rnf123	0.750	0.0126
Gtpbp1	Gtp-Binding Protein 1	0.827	0.0001
Krtcap2	Keratinocyte-Associated Protein 2	1.396	0.0104
Reep5	Receptor Expression-Enhancing Protein 5	0.679	0.0011
Nceh1	Neutral Cholesterol Ester Hydrolase 1	0.814	0.0135
Cotl1	Coactosin-Like Protein	0.770	0.0314
Aass	Alpha-Aminoadipic Semialdehyde Synthase, Mitochondrial	1.296	0.0006
Agmo	Alkylglycerol Monooxygenase	0.727	0.0097
